# Sedation with volatile anaesthetics in intensive care

**DOI:** 10.1016/j.bjae.2023.12.004

**Published:** 2024-01-16

**Authors:** Matthieu Jabaudon, Jean-Michel Constantin

**Affiliations:** 1 CHU Clermont-Ferrand, Clermont-Ferrand, France; 2 iGReD, Université Clermont Auvergne, Clermont-Ferrand, France; 3 Pitié-Salpêtrière Hospital, Sorbonne University, Paris, France

**Keywords:** anaesthetics, inhalation, intensive care unit, sedation

The application of volatile anaesthetics, including isoflurane and sevoflurane, is expanding from the operating theatre into the intensive care unit (ICU). Although commonly used for general anaesthesia, their potential in critical care is attracting increasing interest. This article discusses the theoretical and practical considerations of using volatile anaesthetics for sedation in ICU patients, evaluates the indications and contraindications, weighs the benefits and drawbacks and examines the surveillance measures and potential risks involved. It further addresses implementation hurdles and explores outstanding questions and future challenges. We offer insights into the adoption of volatile anaesthetic sedation into ICU practice, a strategy that may influence outcomes while posing unique challenges that need consideration.

## Pharmacokinetics and pharmacodynamics

The mechanism of action of volatile anaesthetic agents is complicated and not fully understood. They suppress presynaptic excitation and neurotransmitter release, inhibit postsynaptic neurotransmitter activity and exert anti-*N*-methyl-d-aspartic acid (anti-NMDA) and anticonvulsant effects. At higher doses, they can induce isoelectric electroencephalograms with burst suppression.^1^ Furthermore, these agents have bronchodilatory effects, via direct action on bronchial smooth muscle and inhibition of postganglionic vagal impulses.

**Fig 1:**
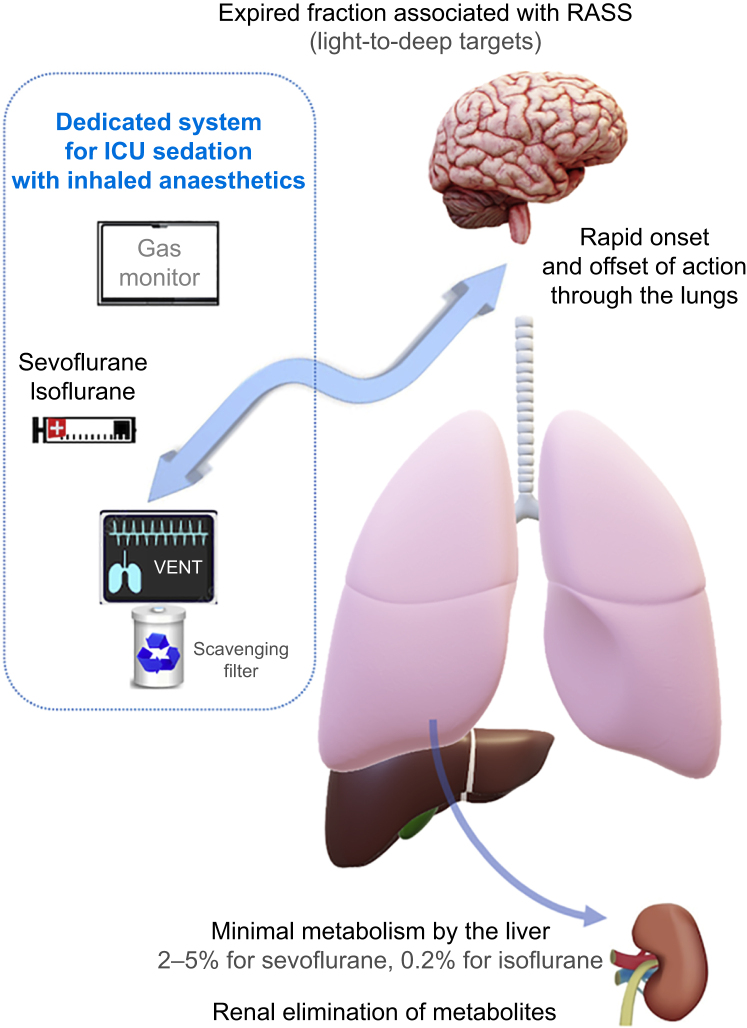
Schematic representation of the use of volatile anaesthetics for sedation in intensive care. RASS, Richmond Agitation Sedation Scale; VENT, ventilator.

Volatile anaesthetics are fast-acting (onset in 1–2 min) and fast-recovery (offset in 4–7 min) drugs that induce a dose-dependent depth of sedation (Fig. 1). This fast offset results from their pulmonary elimination and low degree of hepatic metabolism (2–5% for sevoflurane, 0.2% for isoflurane and 0.02% for desflurane), resulting in no active metabolites (fluorine ions) or any significant change in hepatic or renal laboratory tests in selected ICU patients who were enrolled in published trials.^2^^,^^3^ Although some studies have linked isoflurane or sevoflurane use with a decrease in requirements for opioid and sedative drugs, the mechanisms underlying their analgesic effects remain unclear.^2^^,^^4^ End-tidal anaesthetic concentrations are good surrogates of corresponding brain concentrations and, despite the need to adjust sedation by using validated clinical scores, end-tidal monitoring could serve as a useful method of adjusting sedation according to the patient's needs.^5^

## Delivery devices

Dedicated reflectors have been developed and are now available for use in critically ill patients in the ICU, with two major systems currently available in many countries: the Anaesthetic Conserving Device (Sedaconda ACD-S, Sedana Medical, Danderyd, Sweden) and the Mirus system (TIM GmbH, Koblenz, Germany).

Inhaled sedation devices are inline miniature vaporisers that incorporate humidification and an antiviral filter. These devices are connected between the patient and an ICU ventilator, maintaining up to 90% of the volatile anaesthetic inside the patient. These devices provide excellent humidification and should be used without the addition of a heated humidifier. Although the Sedaconda ACD-S allows for manual titration of sedation through expired fractions of isoflurane or sevoflurane, the Mirus automatically adjusts infusion rates to deliver volatile anaesthetics according to concentration targets and allows use of desflurane in addition to isoflurane or sevoflurane.

## Potential clinical use

### Indications

Historically, volatile anaesthetic agents were first used for patients with severe asthma or bronchospasm and refractory status epilepticus, vaporised through conventional anaesthesia machines in the ICU. They became used increasingly in the ICU in patients with high requirements for sedation such as those with a history of drug addiction or dependency.

Based on two recent surveys in French ICUs, the current preferred indications of sedation with isoflurane or sevoflurane are severe asthma or bronchospasm, acute respiratory distress syndrome (ARDS), and failure of intravenous (i.v.) sedation.^6^^,^^7^ Currently, the Sedaconda ACD-S device is officially approved for delivery of isoflurane for ‘sedation of mechanically ventilated adult patients during intensive care’ in 16 European countries.^8^ This suggests that inhaled anaesthetics (at least isoflurane at the moment) could be considered more widely for this indication. This is in line with Germany's national guidelines, in which using volatile anaesthetics in the ICU can be considered a first-line option in patients undergoing mechanical ventilation and in whom short wake-up times are targeted.^9^

### Contraindications and safety considerations

Patients with a history of malignant hyperthermia or known sensitivity to volatile agents should not receive inhaled sedation.

In patients at risk of increased intracranial pressure, inhalational anaesthetics may be avoided because of their theoretical effects on cerebral blood flow. However, when assessed, the effects of inhaled anaesthetics on intracranial pressure may be less than expected.^10^

Limited data suggest that low-dose isoflurane or sevoflurane has no considerable teratogenic effects in clinical settings of anaesthesia or critical care, in contrast to the incidence of birth defects observed in rats exposed to high, clinically irrelevant doses of isoflurane combined with nitrous oxide. Other potential adverse effects, such as haemodynamic instability or respiratory depression, are dose-dependent and similar to those of i.v. sedatives. Cases of polyuria from nephrogenic diabetes insipidus have been reported with sevoflurane, often after prolonged use at high doses (expired fraction >1.5% for multiple days), for which precise causal mechanisms warrant further investigation.^11^

### Potential advantages

The titratability and pharmacology of volatile anaesthetics enable precise adjustments of the level of sedation, allowing for personalised treatment. Ideally, titration should follow the same clinical analgesia-first sedation scores such as those widely used in ICU patients under i.v. sedation. However, using inhaled sedation also enables monitoring of expired anaesthetic fractions, which could be of particular interest in patients with deeper sedation targets or under neuromuscular block.^5^ Using inhaled sedation for patients in ICU has been associated with wake-up and extubation times approximately half those of i.v. midazolam or propofol in clinical trials.^4^ Of note, specific potential ‘organ-protective’ effects, anti-inflammatory effects, or both of isoflurane or sevoflurane in ICU patients such as in those with ARDS (whether or not related to COVID-19 pneumonitis), sepsis, brain injury, delirium or after resuscitated cardiac arrest have gained recent interest and are currently under evaluation.

### Potential disadvantages

Specialised equipment, including dedicated vaporisers and scavenging systems, is required for the safe delivery of inhaled sedation, which could increase the logistical complexity and cost of inhaled ICU sedation. Environmental pollution is another concern, shared by both volatile and i.v. anaesthetics.^12^ The release of volatile agents may contribute to both global warming and exposure of healthcare providers if not used according to the manufacturers' instructions, which include frequent device replacement and the use of a scavenging system to prevent pollution. Importantly, in order to use volatile anaesthetics effectively and safely in the ICU, it is crucial for healthcare professionals to receive thorough training and education, including on how to recognise and treat malignant hyperthermia. The ICU teams should have a solid understanding of how to handle volatile agents, and grasp the fundamentals of inhaled sedation and use suitable monitoring techniques. It is also essential for standardised protocols to be established locally so that volatile anaesthetics can be used safely and efficiently. However, these protocols should not deviate significantly from those used for i.v. sedation and promote analgesia first; minimise sedation and wakefulness; prevent delirium; and allow early rehabilitation to facilitate weaning from ventilation and ICU discharge.

### Unanswered questions

Despite the growing interest in using volatile anaesthetics in the ICU, several unanswered questions remain. The value of expired fraction monitoring in the management of analgesia-sedation, currently based on clinical scores, requires further investigation. Long-term effects and outcomes associated with volatile-based sedation in ICU patients are also still not fully understood or known. In addition, the potential impact of volatile anaesthetics on cognitive function and delirium in critically ill patients requires further exploration.

## Future challenges

The future of volatile anaesthetic sedation in the ICU faces various challenges. Large-scale studies are needed to further establish the efficacy and safety of volatile anaesthetic use in different patient populations and clinical scenarios in comparison to i.v. sedative agents (Table 1). Research on optimal dosing and duration strategies will further inform ICU-relevant pharmacokinetic models for inhaled anaesthetics. In addition, addressing the environmental impact of volatile agent release and implementing more and more efficient capture, extraction and purification methods in the ICU will be essential to reduce pollution risks, in parallel with recent efforts in the operating theatre.^13^ Education and training programs, and ongoing and future research, will also be crucial in better personalising and delivering inhaled sedation to ICU patients.

**Table 1:** Advantages and disadvantages of volatile anaesthetic sedation compared with intravenous sedation in intensive care.

Aspect	Volatile anaesthetic sedation	Intravenous sedation
Onset and offset of action	Very rapid: onset 1–2 min, offset 4–7 min	Variable
Metabolism and elimination of metabolites	Minimal metabolism through the liver Renal elimination of inactive metabolites	Important liver metabolism and renal elimination of metabolites with risks of accumulation
Sedation targets	Light-to-deep sedation	Variable: light-to-deep sedation for propofol, light sedation only for dexmedetomidine
Titratability	Precise adjustment of sedation depth, expired agent fraction can be monitored	Limited titration capabilities because of longer context-sensitive half-lives, except propofol (but doses >4 mg kg^−1^ h^−1^ associated with propofol infusion syndrome)
Specialised equipment	Requires dedicated vaporisers and scavenging systems	Equipment may be readily available
Environmental pollution	Minimal risk of workplace contamination and exposure Contribution to greenhouse gas emissions	Minimal contribution to greenhouse gas emissions but unmetabolised drugs can seep into the environment with potentially chronic ecotoxic effects

## Conclusions

The use of volatile anaesthetics in the ICU offers advantages such as rapid onset and offset of action enabling precise titration, along with some potential organ-protective effects. However, specific contraindications, specialised equipment requirements and environmental pollution risks should be carefully considered. Vigilant surveillance by educated and trained teams is necessary to mitigate risks and further research is needed to address unanswered questions and potential organ-protective effects of inhaled anaesthetics.

## Declaration of interests

MJ is a principal investigator of the SESAR trial (ClinicalTrials.gov Identifier: NCT04235608), a trial funded by the French Ministry of Health, the European Society of Anaesthesiology, and Sedana Medical. JMC and MJ received fees from Sedana Medical for participation in scientific advisory panels or seminars; MJ received consulting fees from AbbVie. There was no influence from any entities in writing this article.
